# Complete mitochondrial genome and the phylogenetic position of the Lake Eyre hardyhead (*Craterocephalus eyresii*), a freshwater atherinid fish endemic to Lake Eyre Basin, South Australia

**DOI:** 10.1080/23802359.2017.1334527

**Published:** 2017-05-28

**Authors:** Davin H. E. Setiamarga

**Affiliations:** a Department of Applied Chemistry and Biochemistry, National Institute of Technology, Wakayama College, Gobo City, Wakayama, Japan;; b The University Museum, The University of Tokyo, Tokyo, Japan

**Keywords:** Atheriniformes, mitogenome, percomorph teleosts, Lake Eyre hardyhead, South Australia endemic species

## Abstract

The Lake Eyre hardyhead (*Craterocephalus eyresii*) is an endemic freshwater fish living in the Lake Eyre basin in South Australia. Here, I report the full mitogenome description and the phylogenetic position of this species based on the mitogenome phylogenetics. The mitogenome is 16,602 bp-long with the standard 37 genes all included, with a genomic structure typical of a vertebrate mitogenome. A maximum likelihood phylogenetic analysis to confirm this species position was conducted, using a data set including publicly available 28 atherinomorphs, nine percomorphs, and two outgroups. The result confirms *C. eyresii* position's in Atherinoidei. The complete mitogenome data of *C. eyresii* reported here would be useful for further genetics, phylogeography, and phylogenetics studies involving this species.

The Lake Eyre hardyhead, *Craterocephalus eyresii*, is an endemic freshwater species of the family Atherinidae. This species is endemic to the swamps, ponds, lakes, rivers, streams, and lagoons of the Lake Eyre Basin in South Australia. Ecologically, it is an important part of the diet of many local water birds including herons, cormorants, and pelicans (Allen et al. 2002). Interestingly, *C. eyresii* tolerates salinities up to ∼100 ppt (Unmack & Dowling 2010), indicating that it is a secondary freshwater fish species.

Here, I report the full mitochondrial genome (mitogenome) sequence of this species. Tissue sample was collected from an individual specimen from the Ichthyology Collection of the Museum Victoria, Australia (Voucher No. NMV.A. 29477-002). According to the voucher record, the sample was collected in 2006 from Yarra Wurta Springs, 4 km North of Lake Torrens in South Australia. The fish sample was then fixed in ≥90% EtOH. After DNA extraction from the tissue sample, PCR-based mitogenome sequencing using fish versatile primers was conducted in accordance to what was reported previously (Miya & Nishida 1999; Setiamarga et al. 2008). Assembled mitogenome sequence was annotated using the MitoAnnotator on the MitoFish homepage (http://mitofish.aori.u-tokyo.ac.jp/annotation/input.html).

The newly sequenced mitogenome of *Craterocephalus eyresii* was 16,602 bp long (registered to DDBJ). The mitogenomic structure is similar to a typical vertebrate/euteleost mitogenome. (1) There are 13 protein-coding, two rRNA, and 22 tRNA genes. (2) The control region was 880 bp long, situated between the genes tRNA-Pro and tRNA-Phe. (3) Most genes are coded on the H chain, except for ND6 and eight tRNA genes (tRNAPro, tRNAGln, tRNAAla, tRNAAsn, tRNACys, tRNATyr, tRNASer(UCN), and tRNAGlu)); Total GC content of the mitogenome was 47.5%.

A maximum likelihood phylogenetic analysis was done to confirm the phylogenetic position of *C. eyresii* among the atherinomorph fishes. First, publicly available full mitogenome sequences of 12 Atheriniformes, eight Beloniformes, eight Cyprinodontiformes, nine non-atherinomorph percomorphs, and two non-percomorph outgroups were mined from GenBank. Next, using the software RAxML (Stamatakis 2014), I conducted a phylogenetic analysis, which methods are detailed in the legend of Figure 1. The resulting phylogeny was congruent with previous mitogenome (e.g. Kawahara et al. 2008; Setiamarga et al. 2008, 2009) and nuclear gene marker (e.g. Betancur et al. 2013) studies. The monophylies of the order Atheriniformes, superorder Cyprinodontea, and series Atherinomorpha were well supported. The monophylies of the two suborders of Atheriniformes, Atherinopsoidei and Atherinoidei were supported with *C. eyresii* included in the latter suborder.

**Figure 1.**
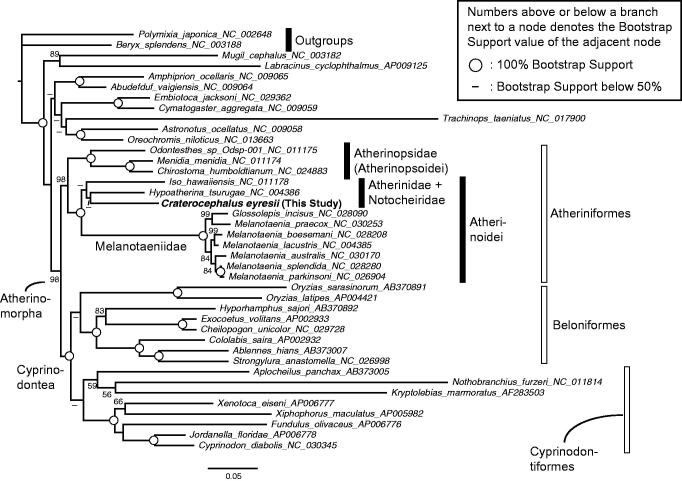
A maximum likelihood (ML) molecular phylogenetic tree, inferred using the program RAxML (Stamatakis 2014). Phylogenetic analyses were conducted on a data matrix (11,080 positions) including all the concatenated nucleotide sequences of the mitogenomes except the third codon positions. The ND6 gene was excluded from the analysis. Gene sequences were aligned individually using the online version of MAFFT under default settings (http://mafft.cbrc.jp/alignment/server/; Katoh & Standley 2013). Aligned sequences were individually edited using the online version of GBlocks using the least stringent settings (http://molevol.cmima.csic.es/castresana/Gblocks_server.html; Castresana 2000). Data partitions were determined using the program PartitionFinder ver. 2 (Lanfear et al. 2017). Partitioned ML analyses were performed with RAxML-GUI ver. 1-5b1 (Silvestro & Michalak 2012), with the GTR + Γ + I nucleotide substitution model (Yang 1994). The rapid bootstrap analyses were conducted with 1000 replications.

The full mitochondrial genome of *C. eyresii* presented here will be useful for future phylogeography and population genetics studies of this Australian endemic species, including to further resolve the interrelationships and vicariance among member species of the “*eyresii* group”, which includes congeners *C. helenae, marianae, munroi, cuneiceps, marjoriae, centralis, amniculus,* and *fluviatilis* besides *C. eyresii* (Unmack & Dowling 2010). Meanwhile, the addition of this full mitogenome sequence will also be useful for future molecular phylogenetics studies of percomorph fishes including, but not limited to, Atherinomorpha and Atheriniformes.
